# Is there variation in utilization of preoperative tests among patients undergoing total hip and knee replacement in the US, and does it affect outcomes? A population-based analysis

**DOI:** 10.1186/s12891-022-05945-y

**Published:** 2022-11-10

**Authors:** Mohamed Mosaad Hasan, Raymond Kang, Jungwha Lee, Matthew D. Beal, Abdalrahman G. Ahmed, Yao Tian, Hassan M. K. Ghomrawi

**Affiliations:** 1 Center for Education in Health Sciences, Institute of Public Health and Medicine, Feinberg School of Medicine, Northwestern University, Chicago, Illinois USA; 2 Center for Community Health, Institute for Public Health and Medicine, Northwestern University, Chicago, Illinois USA; 3 Department of Preventive Medicine, Feinberg School of Medicine, Northwestern University, Chicago, Illinois USA; 4 Center for Epidemiology & Population Health, Institute of Public Health and Medicine, Feinberg School of Medicine, Northwestern University, Chicago, Illinois USA; 5 Hughston Clinics Orthopaedics, Nashville, Tennessee USA; 6 Medical College of Wisconsin, Milwaukee, WI USA; 7 Department of Surgery, Feinberg School of Medicine, Northwestern University, Chicago, Illinois USA; 8 Surgical Outcomes and Quality Improvement Center, Feinberg School of Medicine, Northwestern University, Chicago, Illinois USA; 9 Department of Pediatrics, Feinberg School of Medicine, Northwestern University, Chicago, Illinois USA; 10 Center for Health Services and Outcomes Research, Institute of Public Health and Medicine, Feinberg School of Medicine, Northwestern University, Chicago, Illinois USA; 11 Division of Rheumatology, Department of Medicine, Feinberg School of Medicine, Northwestern University, Chicago, Illinois USA

**Keywords:** Preoperative testing, Total knee replacement, Total hip replacement, MRSA screening, Asymptomatic bacteriuria, Preoperative EKG

## Abstract

**Study objective:**

To describe recent practice patterns of preoperative tests and to examine their association with 90-day all-cause readmissions and length of stay.

**Design:**

Retrospective cohort study using the New York Statewide Planning and Research Cooperative System (SPARCS).

**Setting:**

SPARCS from March 1, 2016, to July 1, 2017.

**Participants:**

Adults undergoing Total Hip Replacement (THR) or Total Knee Replacement (TKR) had a preoperative screening outpatient visit within two months before their surgery.

**Interventions:**

Electrocardiogram (EKG), chest X-ray, and seven preoperative laboratory tests (RBCs antibody screen, Prothrombin time (PT) and Thromboplastin time, Metabolic Panel, Complete Blood Count (CBC), Methicillin Resistance *Staphylococcus Aureus* (MRSA) Nasal DNA probe, Urinalysis, Urine culture) were identified.

**Primary and secondary outcome measures:**

Regression analyses were utilized to determine the association between each preoperative test and two postoperative outcomes (90-day all-cause readmission and length of stay). Regression models adjusted for hospital-level random effects, patient demographics, insurance, hospital TKR, THR surgical volume, and comorbidities. Sensitivity analysis was conducted using the subset of patients with no comorbidities.

**Results:**

Fifty-five thousand ninety-nine patients (60% Female, mean age 66.1+/− 9.8 SD) were included. The most common tests were metabolic panel (74.5%), CBC (66.8%), and RBC antibody screen (58.8%). The least common tests were MRSA Nasal DNA probe (13.0%), EKG (11.7%), urine culture (10.7%), and chest X-ray (7.9%). Carrying out MRSA testing, urine culture, and EKG was associated with a lower likelihood of 90-day all-cause readmissions. The length of hospital stay was not associated with carrying out any preoperative tests. Results were similar in the subset with no comorbidities.

**Conclusions:**

Wide variation exists in preoperative tests before THR and TKR. We identified three preoperative tests that may play a role in reducing readmissions. Further investigation is needed to evaluate these findings using more granular clinical data.

**Supplementary Information:**

The online version contains supplementary material available at 10.1186/s12891-022-05945-y.

## Strengths and limitations of this study

- To our knowledge, this is the first study to use a large administrative database to examine the use and value of preoperative testing.
- We adjusted for confounders, including patient age, gender, race, insurance, and hospital surgical volume. We also controlled for all categories of the Elixhauser comorbidity index.
- We also assessed the relationship between receiving each preoperative test and patient/hospital characteristics.
- We conducted sensitivity analyses, restricting our data to the subset of patients with no Elixhauser comorbidities.
- One of the limitations is that it is a one-state study, and our findings may not represent the preoperative testing practices in all other states.
- Results of these tests were not available in administrative databases, so we do not know whether the clinicians acted on these tests or not.
- Finally, we excluded patients with no reported preoperative visits as we could not rule out whether preoperative tests were conducted in the community or not.

## Introduction

Over a million total hip replacements (THR) and total knee replacements (TKR) are performed in the US each year [1]. Patients undergoing elective total hip replacement (THR) and total knee replacement (TKR) usually undergo a medical evaluation before surgery [2]. The evaluation process involves various laboratory and imaging tests [3]. Clinical guidelines, such as the one set forth by the American Society of Anesthesiologists, recommend a list of tests for THR and TKR; however, given how these guidelines are written, the tests that are ordered for THR and TKR patients are at the providers’ discretion and may result in considerable variability in practice [4].

Preoperative evaluation tests are ubiquitous, but experts disagree regarding the utility of these tests in reducing postoperative healthcare utilization. For example, it is hotly debated whether Methicillin Resistant *Staphylococcus Aureus* (MRSA) testing should be done before TKR to reduce postoperative infection [5]. In fact, performing preoperative tests is supported by limited scientific evidence [3, 6]. In most cases, the results of these tests do not lead to the cancellation of the surgery or a change in the surgical course [6–10]. Nevertheless, these tests involve direct and indirect costs to the patient, healthcare providers, insurance companies, and society at large [6].

Studies from several other surgical fields have demonstrated wide variability in practice [11, 12]; however, little is known about the clinical practice of preoperative testing done on patients before THR and TKR surgery. Therefore, we aimed 1) to examine the current practice patterns of preoperative investigations before elective THR and TKR and 2) to determine the association of nine tests with postoperative outcomes. As a preoperative test is done to detect an abnormality that could affect the outcome, it was essential to examine postoperative outcomes. For example, Complete Blood Count (CBC) determines if anemia exists, as evidence shows that such illness is associated with increased readmission of orthopedic patients within 90 days. Despite the limitations of administrative databases, most readmission performance measures and risk models are founded on administrative rather than clinical databases [13]. Therefore, vetting the association between carrying out the preoperative tests and the outcome measures is justifiably salient. We had two hypotheses: the first hypothesis was wide variability in the preoperative investigations ordered for patients undergoing primary THR and TKR, and the second hypothesis was that few of these tests were associated with postoperative outcomes.

## Methods

### Study cohort

After obtaining the Institutional Review Board (IRB) approval, we utilized the New York Statewide Planning and Research Cooperative System (SPARCS) database to define our cohort. SPARCS is a statewide, all-payer administrative database with unique patient identifiers that collects patient-level data on all inpatient stays. The inclusion criteria were all adult patients who underwent primary THR and TKR and had an outpatient visit with a preoperative screening code (ICD-10-CM code Z01.81x) within two months before their surgery. We retrospectively identified those patients in the SPARCS database between March 1, 2016, and July 1, 2017. We excluded patients who underwent THR and TKR with no information on preoperative visits. To determine if hospitals reporting screening visits were different from those who did not, we compared the characteristics of patients with a preoperative screening visit to patients with no information on preoperative visits using chi-square tests.

### Preoperative tests and outcomes

Nine preoperative investigations recommended by the American Society of Anesthesiologists guidelines [4] were identified using CPT codes and included in our analyses. These included seven laboratory tests (Red Blood Cells (RBCs) antibody screen, coagulation panel, metabolic panel, Complete Blood Count (CBC), Methicillin Resistance *Staphylococcus Aureus* (MRSA) Nasal DNA probe, urinalysis, urine culture), electrocardiogram (EKG), and the chest X-ray. Postoperative outcomes examined in this study were (1) 90-day all-cause readmission (primary outcome) and (2) length of stay (secondary outcome).

### Statistical analysis

We first conducted univariate analyses to describe patients’ sociodemographic and clinical characteristics with a preoperative visit. To investigate hypothesis one, which is to determine the variation in preoperative testing, we compared the proportion of each test among patients who received preoperative screening visits. We used the Marascuillo method to compare the proportion of tests by simultaneously testing the difference between proportions and providing a relative rank [14, 15]. Marascuillo method is a post-hoc test that maintains a prespecified type I error rate and keeps Family-Wise Error Rate (FWER) under control [16]. Compared to other methods like Bonferroni, the Marascuilo method is more conservative [16].

To investigate the second hypothesis, which tests the association between preoperative tests using and postoperative outcomes, we used a hierarchical generalized linear model with a hospital-level random effect were then applied it to determine the association between each preoperative test and 90-day readmission (using logit link function) and hospital length of stay (LOS), identity link function. We used an exchangeable covariance structure to produce smaller estimates and more minor standard errors than other covariance structures. We tested the log transformation of LOS and determined it was unnecessary (Median LOS is three and mean LOS is 2.82). Patients are admitted for a specific procedure (joint replacement), so LOS is not as skewed as all-cause admissions. Regressions were controlled for patient age, gender, race, insurance, and hospital surgical volume. We also controlled for the Elixhauser comorbidities, including each comorbidity as a separate variable. The Elixhauser Comorbidity Index, a validated index based on the International Classification of Diseases (ICD) diagnosis codes, was used to capture the burden of 29 comorbidities known to affect outcomes such as mortality [17]. Each comorbidity in the Elixhauser Comorbidity Index is dichotomous, either present or not [17]. Models were adjusted for patient characteristics (sex, age, race, and ethnicity) and hospital characteristics (hospital volume: i.e., number of primary TKR and THR surgeries per hospital). Hospital volume was represented in the model in quartiles.

Since comorbidities may be driving the preoperative tests ordered for these patients and potential variation in utilization, we conducted sensitivity analyses, restricting our analyses to the subset of patients with no Elixhauser comorbidities. We used SAS software version 9.4 to run this analysis [18]. For preoperative tests associated with lower readmission rates, we calculated the unadjusted admission rates for patients who had undergone preoperative tests and those who did not.

#### Patient and public involvement statement

No patients were involved in setting the research question or the outcome measures, nor were they involved in developing plans for the design or implementation of the study. No patients were asked to advise on the interpretation or writing up of results. There are no plans to disseminate the research results to study participants or the relevant patient community.

## Results

There were 44,964 THR and 65,292 TKR surgeries performed in New York State during the study period. Of those, 55,099 surgeries (50%, 33,930 TKR, and 21,169 THR) had an associated preoperative screening visit code. Compared to those with no preoperative screening visit code, those with the code were more likely to be older, non-Hispanic white, insured by Medicare, and have knee surgery and surgery in hospitals with surgical volume in the 25th to 75th percentile. The prevalence of comorbidities was less than 10% in those with and without preoperative testing, except for the following conditions: hypertension, obesity, chronic pulmonary disease, diabetes, hypothyroidism, deficiency anemias, and fluid and electrolyte disturbance (see Additional file 1: Appendix A).

Of those patients with a preoperative screening visit, 6251 had none of the Elixhauser comorbidities. Compared to patients with no comorbidity, those with one comorbidity or more were more likely to be older and insured by Medicare. The characteristics of all patients and patients without comorbidities are displayed in Table 1.

**Table 1:** Characteristics of total knee and hip replacements patients who have preoperative evaluation visits (primary analysis) and the subset of patients who presented no comorbidities (subset)

	All patients with preoperative tests *N* = 55,099 (primary analysis)	Patients without comorbidities *N* = 6251 (subset)
	n (%)	n (%)
Sex		
Male	33,037 (59.96)	2907 (46.50)
Female	22,062 (40.04)	3344 (53.50)
Age (years)		
8-55	10,407 (18.89)	1759 (28.14)
56-63	10,910 (19.80)	1508 (24.12)
64-69	12,448 (22.59)	1340 (21.44)
70-75	10,304 (18.70)	867 (13.87)
76+	11,030 (20.02)	777 (12.43)
Race		
Non-Hispanic White	42,714 (77.52)	4986 (79.76)
Black	4407 (8.00)	388 (6.21)
Hispanic	4312 (7.83)	466 (7.45)
Asian/Pacific Islander	758 (1.38)	90 (1.44)
Other/Missing race	2908 (5.28)	321 (5.14)
Insurance		
Medicare	28,303 (51.37)	2274 (36.38)
Medicaid	2334 (4.24)	285 (4.56)
Commercial	22,170 (40.24)	3369 (53.90)
Work Comp	1615 (2.93)	230 (3.68)
Other/Unknown	677 (1.23)	93 (1.49)
Hospital Volume		
25th	5546 (10.07)	587 (9.39)
25-50th	9253 (16.79)	1015 (16.24)
50-75th	13,395 (24.31)	1559 (24.94)
75th+	26,905 (48.83)	3090 (49.43)
Surgery Type		
Total Knee Replacement	33,930 (61.58)	3299 (52.78)
Total Hip Replacement	21,169 (38.42)	2952 (47.22)

### Practice patterns of preoperative tests

**Fig. 1:**
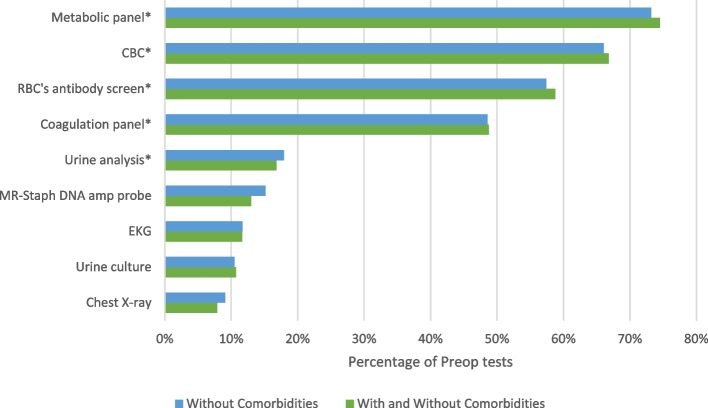
Percentage and ranking of preoperative tests for patients with preoperative visits*

The most prevalent preoperative test was the metabolic panel (74.5% of cases), while the least prevalent was chest X-ray (7.9% of cases), based on the Marascuillo method. Additionally, the proportions of Methicillin Resistance *Staphylococcus Aureus* (MRSA) Nasal DNA probe, EKG, and urine culture were 13, 12, and 11%, respectively, lower than the top five tests (Fig. 1).

There were no differences between THR and TKR patients in the frequency of tests, except TKR patients received slightly more EKG testing (13% TKR vs. 10% THR). However, there were differences based on insurance type and hospital volume. For example, the frequency of common preoperative tests (coagulation panel, metabolic panel, and CBC) in Medicaid patients is the lowest compared to patients with other insurance types. In addition, hospitals with the highest patient volume had lower utilization of less common preoperative tests (urinalysis, urine culture, EKG, and chest X-ray) than lower-volume hospitals.

### Association with outcomes

The readmission rate within 90 days was 8.49%, and the average length of stay was 2.82 days. Three tests were associated with statistically lower readmission rates in the unadjusted analyses. The unadjusted readmission rates for patients with MRSA screening (6.48%), urine culture (5.51%), and EKG (5.62%) were statistically significantly (*P* < 0.0001) lower than those without MRSA screening (8.79%), urine culture (8.85%), or EKG (8.87%). After adjusting for patient and hospital characteristics, regression analysis showed that urine culture (odds ratio (OR) = 0.365, confidence interval (CI) [0.161, 0.829]), EKG (OR = 0.318, CI [0.156, 0.645]), and screening for MRSA (OR = 0.453, CI [0.232, 0.884]) were associated with reduced odds of 90-day readmissions. Still, none of the tests were associated with LOS (Table 2). We observed similar results when we reran the regression models in the subset of patients with no Elixhauser comorbidities (Additional file 2: Appendix B).

**Table 2:** Predictors of 90-day readmission and length of stay

	Total Patients with Preoperative Tests (Main analysis)		Patients Without Comorbidities (Subset)	
	90 Day Readmission OR (95% CI)	LOS Estimate Coefficient (95% CI)	90 Day Readmission OR (95% CI)	LOS Estimate Coefficient (95% CI)
Parameter				
Intercept	0.14 (0.07, 0.28)*	3.14 (2.29, 3.98)*	0.19 (0.09, 0.37)*	3.39 (2.28, 4.49)*
Sex (reference group: male)				
Female	1.15 (0.90, 1.46)	0.24 (0.06, 0.41)*	1.20 (0.95, 1.52)	0.22 (0.03, 0.41)*
Age (years) (reference group: = < 55)				
56-63	0.57 (0.37, 0.87)*	−0.06 (−0.26, 0.14)	0.60 (0.39, 0.94)*	−0.11 (−0.38, 0.16)
64-69	0.54 (0.37, 0.80)*	−0.24 (−0.74, 0.25)	0.49 (0.33, 0.75)*	−0.34 (−1.02, 0.34)
70-75	0.63 (0.38, 1.05)	−0.24 (− 0.81, 0.34)	0.56 (0.33, 0.93)*	− 0.38 (−1.20, 0.44)
76+	1.09 (0.68, 1.75)	0.23 (− 0.43, 0.89)	1.06 (0.64, 1.77)	0.23 (− 0.72, 1.18)
Race (reference group: white)				
Black	0.82 (0.47, 1.46)	1.33 (0.31, 2.36)*	0.96 (0.58, 1.60)	1.44 (0.08, 2.81)*
Hispanic	1.81 (0.95, 3.46)	0.61 (0.40, 0.82)*	1.78 (0.94, 3.35)	0.64 (0.44, 0.85)*
Asian/Pacific Islander	0.51 (0.12, 2.25)	0.60 (−0.20, 1.41)	0.52 (0.13, 2.11)	0.58 (−0.21, 1.38)
Other/Missing race	0.82 (0.41, 1.65)	0.15 (−0.03, 0.33)	0.88 (0.48, 1.63)	0.17 (−0.02, 0.35)
Insurance (reference group: Medicare)				
Medicaid	1.22 (0.49, 3.04)	−0.39 (−1.20, 0.42)	1.16 (0.50, 2.68)	−0.42 (− 1.39, 0.56)
Commercial	0.67 (0.45, 0.99)*	−0.39 (− 1.01, 0.23)	0.59 (0.38, 0.89)*	− 0.51 (− 1.35, 0.32)
Workers Compensation	0.69 (0.29, 1.63)	− 0.11 (− 0.74, 0.51)	0.63 (0.27, 1.48)	− 0.20 (− 1.00, 0.60)
Other/Unknown	0.53 (0.16, 1.77)	− 0.44 (− 1.03, 0.16)	0.63 (0.16, 2.47)	− 0.59 (− 1.44, 0.26)
Hospital volume (reference group: 0-25th)				
25-50th	0.80 (0.42, 1.50)	−0.56 (− 0.86, − 0.26)*	0.77 (0.41, 1.45)	− 0.60 (− 0.94, − 0.26)*
50-75th	1.14 (0.60, 2.16)	− 0.82 (− 1.16, − 0.49)*	1.07 (0.57, 2.02)	−0.87 (− 1.24, − 0.51)*
75th +	0.50 (0.28, 0.87)*	− 0.73 (− 1.13, − 0.33)*	0.47 (0.27, 0.82)*	−0.76 (− 1.22, − 0.29)*
Surgery (reference group: hip)				
Knee	1.71 (1.18, 2.48)*	0.24 (0.08, 0.40)*	1.87 (1.30, 2.69) *	0.23 (0.02, 0.45) *
Preop Test				
RBCs antibody screen	0.91 (0.62, 1.34)	−0.07 (− 0.32, 0.19)	0.91 (0.63, 1.31)	−0.03 (− 0.33, 0.26)
Coagulation panel	1.26 (0.83, 1.94)	0.13 (−0.06, 0.32)	1.19 (0.79, 1.81)	0.11 (−0.11, 0.34)
Metabolic panel	0.85 (0.59, 1.24)	−0.15 (− 0.52, 0.21)	0.87 (0.61, 1.24)	− 0.20 (− 0.63, 0.24)
Complete blood count	1.07 (0.68, 1.68)	− 0.06 (− 0.21, 0.10)	1.10 (0.72, 1.68)	− 0.04 (− 0.19, 0.10)
MR-staph DNA amp probe	0.45 (0.23, 0.88)*	− 0.21 (− 0.57, 0.16)	0.47 (0.24, 0.91)*	− 0.18 (− 0.61, 0.24)
Urinalysis	0.60 (0.34, 1.08)	0.01 (− 0.28, 0.29)	0.61 (0.34, 1.09)	0.02 (− 0.29, 0.34)
Urine culture	0.37 (0.16, 0.83)*	−0.05 (− 0.27, 0.16)	0.36 (0.17, 0.77)*	− 0.10 (− 0.33, 0.14)
EKG	0.32 (0.16, 0.65)*	0.004 (− 0.29,0.30)*	0.35 (0.16, 0.77)*	− 0.01 (− 0.32, 0.29)
Chest X-ray	0.56 (0.29, 1.07)	− 0.09 (− 0.33, 0.16)	0.55 (0.30, 1.03)	− 0.14 (− 0.42, 0.15)

^a^Regressions of 90-day readmission and length of stay are adjusted for the Elixhauser comorbidities; full regression results are shown in the appendix

* *P* < .0001

## Discussion

Patterns of preoperative laboratory tests, EKG, and chest X-rays have not been previously described for the THR and TKR patient population. We aimed to describe these patterns in this patient population and found wide variation in practice, even among patients with no reported comorbidities. Moreover, while none of these tests was associated with length of stay, three tests (MRSA screening, urine culture, and EKG) were associated with lower odds of 90-day readmissions, and the reduction was 55-69%. These findings have important clinical and healthcare policy implications.

In the absence of clear criteria for which tests should be ordered for TKR and THR patients to assess them for surgery, we have shown wide variability in clinical practice. This variability has been reported in studies of other surgical specialties. For example, a national study analyzing preoperative testing for Medicare patients undergoing cataract surgery found that nearly half of patients receive no testing before surgery. In contrast, others received at least one test [12]. In another study of patients who underwent elective non-cardiac surgery, only 38% of patients underwent preoperative evaluation [11]. Multiple factors have been cited in the literature that could explain this variation in the practice of preoperative testing. For example, medicolegal concerns, limited awareness of evidence-based guidelines, concerns about surgical cancellation, practice tradition, and beliefs about surgeons’ expectations may all play a role in ordering unnecessary tests [19]. Variations could also explain this variability among hospitals in the availability of workforce to establish preoperative screening clinics universally and among third-party payers in reimbursing for these tests [3, 20, 21]. On the policy level, bundled payment arrangements could induce this variation as not all arrangements to cover preoperative testing [22]. Further investigation is needed to understand the patient, hospital, geographic, and policy factors contributing to this variation.

Our study showed that three tests, the EKG, the MRSA screening, and the urine culture tests, were associated with substantially lower readmission rates after surgery. These associations were robust, even in the subset of patients with no reported comorbidities. These tests were among the least utilized in this patient population (only 11-13%). Hospitals are under significant financial pressure from insurers to reduce postoperative costs, primarily length of stay (LOS) and readmissions. For example, many hospitals performing THR and TKR are in a bundled payment system, which provides the hospital with a lump sum of money for the inpatient care they offer and all subsequent care within 90 days of surgery [23]. In such an environment, they are always looking for ways to reduce postoperative healthcare utilization and costs [24].

Our analysis showed a reduction in the odds of 90-day readmission by 69% in patients who underwent EKG screening. The American College of Cardiology/American Heart Association (ACC/AHA) guidelines recommend preoperative EKG for patients undergoing intermediate- and high-cardiac risk surgeries [25]. Since the cardiac risk in orthopedic surgeries is intermediate [26], the findings of this study support the ACC/AHA recommendation. As the frequency of abnormal EKG increases with age [27, 28] and most of our study population are older adults, we postulate that the preoperative EKG might have led to changes in anesthesia management which may have subsequently led to better postoperative outcomes [29–31]. However, further investigation is needed to test such hypothesis.

EKG does not influence the cardiac risk but can serve as a baseline for comparison with the postoperative EKG if found abnormal [25]. Adjustments in preoperative testing protocols to include EKG are relatively easy given that this test is low-risk, cheap, and widely available. However, the low rate of EKG may be due to variations among internists who perform preoperative screening in categorizing different orthopedic procedures in the same bucket of intermediate cardiac risk [32]. Further research is warranted to reveal the barriers to performing EKG for TKR and THR patients.

Our study also showed reduced readmissions among patients who had MRSA screening preoperatively. These results add to a growing body of evidence showing the utility of this test in preventing postoperative periprosthetic joint infection (PJI) among TKR and THR patients [5, 33]. Although our study evaluated all-cause readmissions, PJIs constitute about one-third of readmissions within the first few months [34, 35]. PJIs significantly affect patient outcomes and healthcare costs [36–38]. There remains a contentious debate in the orthopedic community regarding the utility of the MRSA test, which may explain the low utilization rate in our cohort [5]. The American Academy of Orthopedic Surgeons (AAOS) has recently called for conducting a multicenter randomized controlled trial to evaluate the value of MRSA screening and decolonization in preventing PJI [39]. While our cohort study was large and population-based, we support more rigorous definitive RCT evidence as suggested by the AAOS to address the conflicting views on this test.

Finally, our study showed that performing a urine culture before surgery was associated with lower odds of 90-day readmission. This test is usually utilized to screen for asymptomatic bacteriuria (ASB). Although screening for ASB is a longstanding practice [40], the limited and conflicting evidence on its benefit could explain the low utilization rate in our cohort [41]. Two meta-analyses suggested an association between ASB and postoperative PJI after arthroplasty [42, 43]. They concluded that preoperative ASB treatment is unnecessary due to the weak microbiological correlation between ASB and PJI [42, 43]. It is important to note that most of the primary studies included in these meta-analyses are non-controlled small cohort studies and may have resulted in such a weak association. In contrast to the prior literature, our study is the first large population-based study, and it found a strong association between urine culture and 90-day readmission. Thus, this study may lay the ground for further investigation of the value of urine culture before TKR and THR.

There are implications to preoperative testing at the patient, provider, and insurer levels. For the patient, the impact of preoperative testing is not limited only to the direct costs of these tests but also involves the indirect costs in the form of travel and missed time from work to have these tests. They also involve additional stress and costs due to the false positives that could lead to further preoperative testing and precautions during and after the surgery. Moreover, these tests include allocating the necessary resources (human and space) to hospitals to ensure patients are fit for surgery associated with high costs. However, identifying preoperative tests associated with lower readmission rates provides an opportunity for patient outcome improvement and cost reduction, which is paramount, especially in the current bundled payment schemes where hospitals shoulder readmission costs. The costs of these tests are minimal compared to the improved outcomes and savings that could be realized from lower readmissions. This paper did not account for the effect of bundled payment arrangement, which may affect preoperative testing practices. We recommend that future research investigates such an effect. As such, these results would be of use to all stakeholders.

## Limitations

This study has some limitations. First, our findings identify the tests associated with better outcomes among patients who underwent surgery. Since we do not observe in this dataset those who did not proceed forward with the surgery based on abnormal tests, we cannot assess the value of these tests. Therefore these findings should not belittle or negate the value of other lab tests in assessing preoperative risks [44]. Second, this is a one-state study. While the state is large, geographically, and socio-demographically diverse, our findings may not represent the preoperative testing practices in all other states. Third, we excluded about half of the patients who underwent TKR and THR surgeries since they did not have associated preoperative screening codes. Our results might not generalize to the whole cohort because their characteristics differ from those with preoperative screening codes. Fourth, we possibly did not capture all the tests that patients underwent, as some tests could have been performed within a short period before the surgery (or for other reasons). Fifth, we did not perform a propensity score analysis as there were too many unobserved factors (both at patients’ and physicians’ levels) which would make matching ineffective. Finally, the results of these tests were not available in the SPARCS database, so we do not know whether the clinicians acted on these tests or not. Hence, we call for more granular clinical studies to confirm the results of this study, and we advocate against increasing the use of EKG, MRSA screening, and urine culture based solely on the results of this study.

## Conclusions

There is wide variation in preoperative testing in TKR and THR. While no preoperative tests are associated with length of stay, MRSA screening, EKG, and urine culture were associated with lower odds of 90-day readmission. These findings call for more research to determine a standardized list of necessary investigations before TKR and THR that should be applied to all patients, resulting in better stewardship of these tests and more prudent and systematic use.

## Supplementary Information

**Additional file 1:** **Table A1.** 2016-2017 Knee and Hip replacements for patients with and without information on pre-operative screening visit. Table A2. Comorbidity Profile of those with and without preoperative testing information.

**Additional file 2.** Multi-variable Regression with a Hospital-level Random Effect for Patients without comorbidities.

## Data Availability

The data that support the findings of this study are available from the New York State Department of Health (NYSDOH) but restrictions apply to the availability of these data, which were used under license for the current study, and so are not publicly available. Data are however available from the authors upon reasonable request and with permission of the New York State Department of Health (NYSDOH).
