# Supplementary material for: Is there variation in utilization of preoperative tests among patients undergoing total hip and knee replacement in the US, and does it affect outcomes? A population-based analysis
Source: BMC Musculoskelet Disord. 2022 Nov 10;23:972. doi: 10.1186/s12891-022-05945-y (PMC9647906; doi:10.1186/s12891-022-05945-y)
Supplement: Supplementary file 1 — Additional file 1: Table A1. 2016-2017 Knee and Hip replacements for patients with and without information on pre-operative screening visit. Table A2. Comorbidity Profile of those with and without preoperative testing information. [file 12891_2022_5945_MOESM1_ESM.docx]

**Appendix A**

**Table A1.** 2016-2017 Knee and Hip replacements for patients with and without information on pre-operative screening visit

|  | Preop clearance visit  N= 55099 | No information on Preop clearance visit  N= 55157 | P-Value |
| --- | --- | --- | --- |
|  | % | % | 0.1239 |
| Male | 59.96 | 60.48 |  |
| Female | 40.04 | 39.51 |  |
| Age |  |  | <.0001 |
| 1) 8-55 | 18.89 | 20.66 |  |
| 2) 56-63 | 19.80 | 21.62 |  |
| 3) 64-69 | 22.59 | 21.30 |  |
| 4) 70-75 | 18.70 | 17.37 |  |
| 5) 76+ | 20.02 | 19.05 |  |
| Race |  |  | <.0001 |
| Non-Hispanic White | 77.52 | 70.78 |  |
| 2) Black | 8.00 | 11.36 |  |
| 3) Hispanic | 7.83 | 6.82 |  |
| 4) Asian/PI | 1.38 | 2.42 |  |
| 5) Other/MR | 5.28 | 8.62 |  |
| Insurance |  |  | <.0001 |
| Medicare | 51.37 | 46.77 |  |
| 2) Medicaid | 4.24 | 3.22 |  |
| 3) Commercial | 40.24 | 45.75 |  |
| 4) Work Comp | 2.93 | 3.06 |  |
| 5) Other/Unknown | 1.23 | 1.21 |  |
| Hospital Volume |  |  | <.0001 |
| 1) 25^th^ | 10.07 | 11.03 |  |
| 2) 25-50^th^ | 16.79 | 14.65 |  |
| 3) 50-75^th^ | 24.31 | 23.49 |  |
| 4) 75th+ | 48.83 | 50.83 |  |
| Surgery Type |  |  | <.0001 |
| Total Knee Replacement | 61.58 | 56.86 |  |
| Total Hip Replacement | 38.42 | 43.14 |  |
| Pre-op Screen Test |  |  |  |
| RBCs antibody screen | 58.76 | n/a |  |
| Coagulation panel | 48.74 | n/a |  |
| Metabolic panel | 74.51 | n/a |  |
| CBC | 66.81 | n/a |  |
| MR-staph DNA amp probe | 12.99 | n/a |  |
| urinalysis | 16.81 | n/a |  |
| urine culture | 10.70 | n/a |  |
| EKG | 11.68 | n/a |  |
| chest X-ray | 7.90 | n/a |  |

n/a: not applicable, PI: Pacific Islander, MR: multiple race, EKG (electrocardiogram), MR-staph DNA amp probe (Methicillin Resistance Staphylococcus Aureus), CBC (Complete Blood).

**Table A2.** Comorbidity Profile of those with and without preoperative testing information

|  | Preoperative screening visit | No information on preoperative screening visit |  |
| --- | --- | --- | --- |
| Number | 55099 | 55157 | P-value |
| Comorbidity | % | % |  |
| Acquired immune deficiency syndrome | 0.1% | 0.1% | 0.7259 |
| Alcohol abuse | 1.5% | 1.6% | 0.1314 |
| Chronic blood loss anemia | 0.9% | 0.6% | <.0001 |
| Chronic pulmonary disease | 18.7% | 16.1% | <.0001 |
| Coagulopathy | 3.6% | 2.5% | <.0001 |
| Congestive heart failure | 2.5% | 2.8% | 0.0026 |
| Deficiency Anemias | 11.1% | 9.0% | <.0001 |
| Depression | 13.8% | 11.4% | <.0001 |
| Diabetes w/ chronic complications | 5.2% | 4.5% | <.0001 |
| Diabetes w/o chronic complications | 15.4% | 13.7% | <.0001 |
| Drug abuse | 1.1% | 1.2% | 0.1171 |
| Fluid and electrolyte disorders | 10.1% | 10.4% | 0.062 |
| Hypertension | 65.4% | 60.0% | <.0001 |
| Hypothyroidism | 16.7% | 14.4% | <.0001 |
| Liver disease | 1.8% | 1.9% | 0.4468 |
| Lymphoma | 0.4% | 0.3% | 0.7157 |
| Metastatic cancer | 0.4% | 0.6% | <.0001 |
| Obesity | 33.2% | 30.7% | <.0001 |
| Other neurological disorders | 4.7% | 4.4% | 0.0222 |
| Paralysis | 0.6% | 0.8% | 0.0013 |
| Peptic ulcer Disease x bleeding | 0.6% | 0.5% | 0.2372 |
| Peripheral vascular disease | 2.4% | 1.8% | <.0001 |
| Psychoses | 1.8% | 1.8% | 0.6988 |
| Pulmonary circulation disease | 0.3% | 0.3% | 0.3485 |
| Renal failure | 5.7% | 5.5% | 0.2642 |
| Rheumatoid arthritis/collagen vas | 4.7% | 4.2% | <.0001 |
| Solid tumor w/out metastasis | 0.9% | 0.8% | 0.0891 |
| Valvular disease | 3.0% | 2.8% | 0.0844 |
| Weight loss | 0.5% | 0.9% | <.0001 |
