# Supplementary material for: Is there variation in utilization of preoperative tests among patients undergoing total hip and knee replacement in the US, and does it affect outcomes? A population-based analysis
Source: BMC Musculoskelet Disord. 2022 Nov 10;23:972. doi: 10.1186/s12891-022-05945-y (PMC9647906; doi:10.1186/s12891-022-05945-y)
Supplement: Supplementary file 2 — Additional file 2. Multi-variable Regression with a Hospital-level Random Effect for Patients without comorbidities. [file 12891_2022_5945_MOESM2_ESM.docx]

Appendix B. Multi-variable Regression with a Hospital-level Random Effect for Patients without comorbidities

|  | **90 Day Readmission** | **LOS** |
| --- | --- | --- |
|  | OR (95% CI) | Coefficient (95% CI) |
| Intercept | 0.187 (0.094, 0.372) | 3.388 (2.284, 4.492) |
| Sex |  |  |
| F | 1.203 (0.953, 1.518) | 0.220 (0.027, 0.413)* |
| 2) 56-63 | 0.604 (0.388, 0.939)* | -0.112 (-0.384, 0.160)* |
| 3) 64-69 | 0.494 (0.325, 0.753)* | -0.339 (-1.022, 0.344) |
| 4) 70-75 | 0.557 (0.334, 0.931)* | -0.376 (-1.195, 0.443) |
| 5) 76+ | 1.060 (0.636, 1.765) | 0.233 (-0.717, 1.183) |
| 2) Black | 0.963 (0.579, 1.601) | 1.443 (0.079, 2.807) |
| 3) Hispanic | 1.778 (0.944, 3.350) | 0.641 (0.435, 0.847)* |
| 4) Asian/PI | 0.520 (0.128, 2.109) | 0.583 (-0.210, 1.377) |
| 5) Other/MR | 0.880 (0.476, 1.627) | 0.168 (-0.018, 0.354)* |
| 2) Medicaid | 1.155 (0.497, 2.682) | -0.418 (-1.391, 0.556) |
| 3) Commercial | 0.585 (0.384, 0.890)* | -0.512 (-1.347, 0.323) |
| 4) Work Comp | 0.631 (0.270, 1.476) | -0.199 (-1.000, 0.603) |
| 5) Other/Unknown | 0.626 (0.158, 2.474) | -0.587 (-1.436, 0.262) |
| 2) 25-50th | 0.772 (0.411, 1.450) | -0.601 (-0.940, -0.262)* |
| 3) 50-75th | 1.069 (0.567, 2.016) | -0.873 (-1.239, -0.506) |
| 4) 75th+ | 0.469 (0.268, 0.821) | -0.756 (-1.223, -0.289) |
| 1) Knee | 1.869 (1.297, 2.694)* | 0.233 (0.020, 0.447)* |
| RBCs antibody screen | 0.910 (0.632, 1.308) | -0.034 (-0.325, 0.258)* |
| Coagulation panel | 1.192 (0.785, 1.809) | 0.114 (-0.108, 0.337)* |
| Metabolic panel | 0.869 (0.610, 1.240) | -0.196 (-0.632, 0.240)* |
| CBC | 1.101 (0.721, 1.681) | -0.042 (-0.187, 0.103)* |
| MRSA DNA amp probe | 0.467 (0.240, 0.905)* | -0.184 (-0.607, 0.239)* |
| urinalysis | 0.612 (0.344, 1.086) | 0.023 (-0.294, 0.341)* |
| urine culture | 0.362 (0.170, 0.771)* | -0.097 (-0.332, 0.138)* |
| EKG | 0.350 (0.158, 0.773)* | -0.013 (-0.319, 0.292)* |
| Chest x-ray | 0.551 (0.295, 1.029) | -0.139 (-0.424, 0.147)* |

* Statistically significant

PI: Pacific Islander, MR: multiple race, EKG (electrocardiogram), MR-staph DNA amp probe (Methicillin Resistance Staphylococcus Aureus), CBC (Complete Blood
